# Method to estimate relative risk using exposed proportion and case group data

**DOI:** 10.1038/s41598-017-02302-1

**Published:** 2017-05-18

**Authors:** Yoichi Yada

**Affiliations:** Division of Pharmacology, Department of Biomedical Sciences, Nihon University School of Medicine, 30-1 Oyaguchi-kamicho, Itabashi City, Tokyo 173-8610 Japan

## Abstract

A change in risk of an event occurring, which is affected with a factor, is a common issue in many research fields, and relative risk is widely used because of intuitive interpretation. Estimating relative risk has required data from two follow-up groups and can thus be cost and time consuming. Subjects for whom an event occurred (case group) are often observed but generally analyzed in comparison to those for whom an event did not (control group); however, estimating relative risk using case group data without approximation is hindered. In this study, an obstacle to estimate relative risk using case control data is clarified as a mathematical expression and a new equation to estimate relative risk using the exposed proportion and case group data is proposed. The proposed equation is derived without using the Bayesian methods. A method to estimate the confidence interval for the proposed estimator is also provided. The usefulness of the proposed equation, which requires neither control nor follow-up groups, is demonstrated for both theoretical and real-life examples.

## Introduction

A change in risk of an event occurring associated with exposure to a factor is generally studied in many fields, such as medicine and social science^1, 2^. Relative risk (*RR*), also known as “rate ratio”, is widely used as a measure of association and can be interpreted intuitively^3, 4^ because of its simple definition:

1 $$RR\equiv \frac{{\pi }_{1}}{{\pi }_{0}},$$

where *π*_1_ and *π*_0_ are the probabilities of an event occurring (i.e., risks) for subjects exposed and unexposed to a factor. Estimating *RR* requires the estimators of both *π*_1_ and *π*_0_, such as the prevalence or cumulative incidence rate.

The probability estimators can be calculated using existing data of large-scale epidemiological studies or should be obtained from a smaller study designed for the estimation. Let *N* be the total number of subjects to be studied, such as population, and *N*_1_ and *N*_0_ be the exposed and unexposed parts of *N*. The *N*_1_ is written as

2 $${N}_{1}=N-{N}_{0}=E\cdot N,$$

where *E* is the exposed proportion. The probabilities of an event occurring can be written as

3 $${\pi }_{1}=\frac{{N}_{11}}{{N}_{1}}\,{\rm{and}}\,{\pi }_{0}=\frac{{N}_{01}}{{N}_{0}},$$

where *N*_11_ and *N*_01_ are the numbers of subjects for whom an event occurred among *N*_1_ and *N*_0_. When *p*_1_ and *p*_0_ are the estimators of *π*_1_ and *π*_0_, they should be defined as

4 $${p}_{1}\equiv \frac{{n}_{11}}{{n}_{1}}\,{\rm{and}}\,{p}_{0}\equiv \frac{{n}_{01}}{{n}_{0}},$$

where *n*_1_ and *n*_0_ are the observed numbers of exposed and unexposed subjects and *n*_11_ and *n*_01_ are the numbers of subjects for whom the event occurred among *n*_1_ and *n*_0_. Thus, *eRR*, which is defined as

5 $$eRR\equiv \frac{{n}_{11}\cdot {n}_{1}^{-1}}{{n}_{01}\cdot {n}_{0}^{-1}},$$

is used as the estimator of relative risk. The groups of *n*_11_ and *n*_01_ can be found in groups of exposed and unexposed subjects, who were followed to the event occurring (called “cohort”). However, appropriate cohorts may be occasionally found in epidemiological survey results or should be obtained from a fresh study designed for the purpose (i.e., cohort study).

Unfortunately, few existing results provide appropriate cohorts and long-term observations of cohorts, for example, over several years or decades, are likely to be costly and time consuming, and thus, estimating relative risk can be burdensome for researchers. Meanwhile, because case groups are commonly observed, studies comparing them to a control group (case control study) and estimating the change in risk tend to be less costly and time consuming. Although a case control study is often conducted, estimating relative risk using case control data is hindered. To demonstrate, let *m*_1_ and *m*_0_ be the numbers of observed subjects in a case group and control group and *m*_11_ and *m*_01_ be the numbers of exposed subjects in the case and control groups (see Table 1). When *meRR* is defined similarly to the estimator of relative risk as

6 $$meRR\equiv \frac{{m}_{11}\cdot {({m}_{11}+{m}_{10})}^{-1}}{({m}_{1}-{m}_{11})\cdot {({m}_{1}-{m}_{11}+{m}_{0}-{m}_{10})}^{-1}},$$

*meRR* may be misused as an estimator of relative risk but will largely vary with observing conditions that researchers can designate, such as the size of *m*_1_. Moreover, researchers cannot perceive the effects of those observing conditions. Thus, *meRR* is not appropriate for the estimation. Although this obstacle for estimating relative risk caused by observation is well known to epidemiologists^1^, few studies have clarified the effects of observing conditions as a mathematical expression.

**Table 1:** Contingency tables for all subjects, cohort, case control, and random sample data.

	All subjects			Cohort data			Case control data		Random sample data
	Occurred		Total	Occurred		Total	Case group	Control group	
	Yes	No		Yes	No				
Exposed	*N*_11_	*N*_1_–*N*_11_	*N*_1_	*n*_11_	*n*_1_–*n*_11_	*n*_1_	*m*_11_	*m*_10_	*l*_1_
Unexposed	*N*_01_	*N*_0_–*N*_01_	*N*_0_	*n*_01_	*n*_0_–*n*_01_	*n*_0_	*m*_1_–*m*_11_	*m*_0_–*m*_10_	*l*–*l*_1_
Total			*N*				*m*_1_	*m*_0_	*l*

“Subjects”(*N*) comprise “Exposed”(*N*_1_) and “Unexposed”(*N*_0_), both of which include subjects for whom an event occurred (*N*_11_ and *N*_01_). Both of exposed and unexposed cohort (*n*_1_ and *n*_0_) have subjects for whom the event occurred (*n*_11_ and *n*_01_). Exposed subjects (*m*_11_ and *m*_10_) can be found in both of case and control group (*m*_1_ and *m*_0_). Exposed subjects (*l*_1_) can be found in a random sample of the whole subjects (*l*).

According to Cornfield (1951), relative risk can be approximated using an odds ratio (*OR*)^5^, which is defined as

7 $$OR\equiv \frac{{\pi }_{1}\cdot {\mathrm{(1}-{\pi }_{1})}^{-1}}{{\pi }_{0}\cdot {\mathrm{(1}-{\pi }_{0})}^{-1}}=\frac{{N}_{11}\cdot {({N}_{1}-{N}_{11})}^{-1}}{{N}_{01}\cdot {({N}_{0}-{N}_{01})}^{-1}},$$

when *π*_0_ is small (so-called “rare disease assumption”). Thus, the estimator of *OR* (*eOR*), which is defined as

8 $$eOR\equiv \frac{{m}_{11}\cdot ({m}_{0}-{m}_{10})}{{m}_{10}\cdot ({m}_{1}-{m}_{11})},$$

is often computed instead of estimating relative risk. However, *OR* always overstates the association and the divergence of overstatement depends on *RR* or *π*_0_^6, 7^ and thus, using *eOR* may be misleading.

In addition, some study designs that reduce costs and estimate relative risk were proposed^8–10^, although they still require cohorts or the likes. Few studies have focused on deriving the above equations. Zhang and Yu (1998) proposed an equation that can compute relative risk from the odds ratio^11^ as follows:

9 $$RR=\frac{OR}{1+{\pi }_{0}\cdot (OR-\mathrm{1)}}=OR+{\pi }_{1}\cdot \mathrm{(1}-OR\mathrm{).}$$

This equation served as a new method to estimate relative risk using case control data; however, the estimator of *π*_0_ or *π*_1_ is still required to perform the calculation.

Other than above, the Bayesian methods also provide an equation of relative risk. When P_o_ and P_e_ are the probabilities of finding subjects for whom an event occurred and who were exposed to a factor, the Bayes’ theorem^12^ can be written as

10 $${\pi }_{1}=\frac{{{\rm{P}}}_{{\rm{eo}}}\cdot {{\rm{P}}}_{{\rm{o}}}}{{{\rm{P}}}_{{\rm{e}}}},$$

where P_eo_ is the probability of finding subjects who were exposed to a factor among subjects for whom an event occurred. Because *π*_0_ can be written as

11 $${\pi }_{0}=\frac{\mathrm{(1}-{{\rm{P}}}_{{\rm{eo}}})\cdot {{\rm{P}}}_{{\rm{o}}}}{1-{{\rm{P}}}_{{\rm{e}}}},$$

then *RR* is

12 $$RR=\frac{1-{{\rm{P}}}_{{\rm{e}}}}{{{\rm{P}}}_{{\rm{e}}}}\cdot \frac{{{\rm{P}}}_{{\rm{eo}}}}{1-{{\rm{P}}}_{{\rm{eo}}}}\mathrm{.}$$

However, because P_eo_ and P_e_ will vary depending on methods of observation, precise estimation with using this equation should require follow-up data of all subjects or a carefully collected random sample of that. Moreover, because of difference in probability definitions, such as using “the probability of finding exposed subjects” rather than “the exposed proportion”, there is resistance toward the Bayesian methods among some researchers, such as traditional statisticians.

This study illustrates an obstacle, which prevent relative risk from being estimated using case control data, as a mathematical expression of inconsistency in the observations and proposes a new equation to estimate relative risk, which requires case group data and the exposed proportion. The proposed equation is derived without the Bayesian methods, and do not require the probability estimators; that is, neither control groups nor cohorts are needed. Theoretical and real-life examples that demonstrate validity and wide applicability of the proposed equation are also provided.

## Results

To clarify an obstacle in estimating relative risk using case control data and derive an equation to estimate relative risk, let us introduce a proportion of observed subjects among all subjects of interest (hereinafter, “observed proportion”). For example, the number of observed individuals exposed to a factor divided by the exposed population constitutes the observed proportion of exposed individuals. As a expression, the observed proportion is the same as “the sampling proportion”, which is the proportion of a sample among all subjects of interest. However, the observed proportion cannot be estimated while the sampling proportion can be even assigned by researchers.

In cohort studies, the observed proportions can be defined as follows:

13 $$O{P}_{\exp }\equiv \frac{{n}_{1}}{{N}_{1}}=\frac{{n}_{11}}{{N}_{11}}+{d}_{\exp }$$

and

14 $$O{P}_{{\rm{unexp}}}\equiv \frac{{n}_{0}}{{N}_{0}}=\frac{{n}_{01}}{{N}_{01}}+{d}_{{\rm{unexp}}},$$

where *OP*_exp_ and *OP*_unexp_ are the observed proportions of exposed and unexposed subjects and *d*_exp_ and *d*_unexp_ are constants. Cohort studies must be designed as follows:

15 $$\frac{{n}_{11}}{{n}_{1}}=\frac{{N}_{11}}{{N}_{1}}\,(\iff \frac{{n}_{1}}{{N}_{1}}=\frac{{n}_{11}}{{N}_{11}})$$

and

16 $$\frac{{n}_{01}}{{n}_{0}}=\frac{{N}_{01}}{{N}_{0}}\,(\iff \frac{{n}_{0}}{{N}_{0}}=\frac{{n}_{01}}{{N}_{01}}),$$

such that *d*_exp_ and *d*_unexp_ are sufficiently small to be ignored. Inserting equations (13) and (14) into equation (5), we obtain

17 $$eRR=\frac{\{(O{P}_{\exp }-{d}_{\exp })\cdot {N}_{11}\}\cdot {(O{P}_{\exp }\cdot {N}_{1})}^{-1}}{\{(O{P}_{{\rm{unexp}}}-{d}_{{\rm{unexp}}})\cdot {N}_{01}\}\cdot {(O{P}_{{\rm{unexp}}}\cdot {N}_{0})}^{-1}}\mathrm{.}$$

When *d*_exp_ = 0 and *d*_unexp_ = 0,

18 $$eRR=\frac{{N}_{11}\cdot {N}_{1}^{-1}}{{N}_{01}\cdot {N}_{0}^{-1}}=\frac{{\pi }_{1}}{{\pi }_{0}}\mathrm{.}$$

Therefore, *eRR* can be used to estimate the relative risk in cohort studies.

In case control studies, the observed proportions may be defined as follows:

19 $$O{P}_{{\rm{case}}}\equiv \frac{{m}_{11}}{{N}_{11}}=\frac{{m}_{1}-{m}_{11}}{{N}_{01}}+{d}_{{\rm{case}}}$$

and

20 $$O{P}_{{\rm{cont}}}\equiv \frac{{m}_{10}}{{N}_{1}-{N}_{11}}=\frac{{m}_{0}-{m}_{10}}{{N}_{0}-{N}_{01}}+{d}_{{\rm{cont}}},$$

where *OP*_case_ and *OP*_cont_ are the observed proportions of case group and control group and *d*_case_ and *d*_cont_ are constants. Case control studies must be designed as

21 $$\frac{{m}_{11}}{{m}_{1}-{m}_{11}}=\frac{{N}_{11}}{{N}_{01}}\,(\iff \frac{{m}_{11}}{{N}_{11}}=\frac{{m}_{1}-{m}_{11}}{{N}_{01}})$$

and

22 $$\frac{{m}_{10}}{{m}_{0}-{m}_{10}}=\frac{{N}_{1}-{N}_{11}}{{N}_{0}-{N}_{01}}\,(\iff \frac{{m}_{10}}{{N}_{1}-{N}_{11}}=\frac{{m}_{0}-{m}_{10}}{{N}_{0}-{N}_{01}}),$$

such that *d*_case_ and *d*_cont_ should be sufficiently small to be ignored. Substituting equations (19) and (20) in equation (8), we obtain

23 $$eOR=\frac{O{P}_{{\rm{case}}}\cdot {N}_{11}\cdot \{(O{P}_{{\rm{cont}}}-{d}_{{\rm{cont}}})\cdot ({N}_{0}-{N}_{01})\}}{O{P}_{{\rm{cont}}}\cdot ({N}_{1}-{N}_{11})\cdot \{(O{P}_{{\rm{case}}}-{d}_{{\rm{case}}})\cdot {N}_{01}\}}\mathrm{.}$$

When *d*_case_ = 0 and *d*_cont_ = 0,

24 $$eOR=\frac{{N}_{11}\cdot {({N}_{1}-{N}_{11})}^{-1}}{{N}_{01}\cdot {({N}_{0}-{N}_{01})}^{-1}}=\frac{{\pi }_{1}\cdot {\mathrm{(1}-{\pi }_{1})}^{-1}}{{\pi }_{0}\cdot {\mathrm{(1}-{\pi }_{0})}^{-1}}\mathrm{.}$$

Therefore, *eOR* can be used to estimate the odds ratio.

However, inserting equations (19) and (20) into equation (6), we must obtain

25 $$meRR=\frac{O{P}_{{\rm{case}}}\cdot {N}_{11}\cdot {\{O{P}_{{\rm{case}}}\cdot {N}_{11}+O{P}_{{\rm{cont}}}\cdot ({N}_{1}-{N}_{11})\}}^{-1}}{O{P}_{{\rm{case}}}\cdot {N}_{01}\cdot {\{O{P}_{{\rm{case}}}\cdot {N}_{01}+O{P}_{{\rm{cont}}}\cdot ({N}_{0}-{N}_{01})\}}^{-1}}$$

when *d*_case_ = 0 and *d*_cont_ = 0. Thus assuming *OP*_case_ is equivalent to *OP*_cont_, *meRR* can estimate the relative risk. Unfortunately, the equivalence of *OP*_case_ and *OP*_cont_ cannot be estimated but must be tested.

Equation (25) is a mathematical expression that illustrates an obstacle to estimate relative risk using case control data. Thus, excluding both *OP*_case_ and *OP*_cont_ would clearly remove this obstacle in estimating relative risk.

Here, let us focus on the exposure odds, which is the ratio of exposed subjects to unexposed ones. Let *EOC* be the exposure odds in a case group and defined as

26 $$EOC\equiv \frac{{m}_{11}}{{m}_{1}-{m}_{11}}\mathrm{.}$$

Inserting equation (19) into equation (26) leads

27 $$EOC=\frac{O{P}_{{\rm{case}}}\cdot {N}_{11}}{(O{P}_{{\rm{case}}}-{d}_{{\rm{case}}})\cdot {N}_{01}}\mathrm{.}$$

When *d*_case_ = 0, substituting equations (2) and (3) into equation (27) leads

28 $$EOC=\frac{E}{1-E}\cdot \frac{{\pi }_{1}}{{\pi }_{0}}\mathrm{.}$$

Assume that a random sample is selected from all subjects and *eE* is the proportion of exposed subjects among the sample. Thus, *eE* can be written as

29 $$eE\equiv \frac{{l}_{1}}{l},$$

where *l* is the size of a random sample and *l*_1_ is the number of exposed subjects among the sample. The observed proportion of a random sample (that is, the sampling proportion) may be defined as

30 $$O{P}_{{\rm{sample}}}\equiv \frac{l}{N}=\frac{{l}_{1}}{{N}_{1}}+{d}_{{\rm{sample}}},$$

where *d*_sample_ is a constant. Inserting equation (30) into equation (29),

31 $$eE=\frac{(O{P}_{{\rm{sample}}}-{d}_{{\rm{sample}}})\cdot {N}_{1}}{O{P}_{{\rm{sample}}}\cdot N}$$

Because the random sampling should provide

32 $$\frac{{N}_{1}}{N}=\frac{{l}_{1}}{l},$$

then *d*_sample_ is sufficiently small to be ignored. When *d*_sample_ = 0, inserting equation (2) into equation (31) leads

33 $$eE=E\mathrm{.}$$

Thus, let *PRR* be defined as

34 $$PRR\equiv \frac{l-{l}_{1}}{{l}_{1}}\cdot \frac{{m}_{11}}{{m}_{1}-{m}_{11}}\mathrm{.}$$

Substituting equations (26) and (29) into equation (34) leads

35 $$PRR=\frac{1-eE}{eE}\cdot EOC\mathrm{.}$$

Both *d*_case_ and *d*_sample_ should be sufficiently small to be ignored when a random sample is selected from all subjects of whom a case group represents an event-occurring part. When *d*_case_ = 0 and *d*_sample_ = 0, combining equations (28), (33), and (35), we must obtain

36 $$PRR=\frac{1-E}{E}\cdot (\frac{E}{1-E}\cdot \frac{{\pi }_{1}}{{\pi }_{0}})=\frac{{\pi }_{1}}{{\pi }_{0}}\mathrm{.}$$

Therefore, *PRR* must be an estimator of relative risk when subjects among whom a case group is observed and subjects from whom a random sample is selected are the same.

This estimator is computed from the exposure odds in a case group and those in all subjects to be studied, and thus, no control group is required. In addition, the estimation is performed without a cohort.

Equation (34) is quite similar to equation (12), but note that *PRR* was derived without using the Bayesian methods and can be applicable to more general data: data of a case group and a random sample.

Therefore, by considering the observed proportions, an observational inconsistency preventing relative risk from being estimated in the case control studies was clarified as a mathematical expression, and a new equation to estimate relative risk using the exposed proportion and a case group was proposed; the proposed equation requires neither control groups nor cohorts.

### Application to Model Data

Suppose the probabilities of disease Y developing among people exposed and unexposed to chemical compound X are 0.03 and 0.01 (i.e., relative risk is 3).

When the proportion of exposed people in a city, which has a population of 100000, is 30%, researchers should observe the following data: 30 patients are found among 1000 exposed participants and 10 patients among 1000 unexposed participants during a follow-up period; 180 exposed patients are observed in a case group of 320 and 97 exposed participants are observed in a control group of 328; and 300 exposed people are found in a random sample of 1000 participants (see Table 2). The observed proportions of the case and control groups, which are unavailable for the researchers, are then 1/5 and 1/300.

**Table 2:** Model data: population, cohort, case control, and census data.

	Population			Cohort data			Case control data		Random sample data
	Developed		Total	Developed		Total	Case Group	Control Group	
	Yes	No		Yes	No				
Exposed	900	29100	30000	30	970	1000	180	97	300
Unexposed	700	69300	70000	10	990	1000	140	231	700
Total			100000				320	328	1000

This city, which has a population of 100000, and 30000 individuals exposed to X, includes 900 exposed and 700 unexposed patients who developed Y. Accordingly, 30 and 10 patients should be found when 1000 exposed and 1000 unexposed participants have been observed as cohorts; 180 patients and 97 participants should have been exposed when a case group of 320 and a control group of 328 are observed; and 300 exposed people should be found when 1000 individuals are randomly observed.

Thus, estimating relative risk from cohort data must be

37 $$eRR=\frac{30/1000}{10/1000}=\mathrm{3.00.}$$

Estimating odds ratio from case-control data is

38 $$eOR=\frac{180\times 231}{140\times 97}=3.06$$

and *meRR* should be

39 $$meRR=\frac{180/\mathrm{(180}+\mathrm{97)}}{140/\mathrm{(140}+\mathrm{231)}}=\mathrm{1.72.}$$

Finally, the proposed estimator *PRR* can be computed as

40 $$PRR=\frac{1000-300}{300}\times \frac{180}{140}=\mathrm{3.00.}$$

Note that the proposed equation will estimate the relative risk as precisely as the estimation in a cohort study but does not require follow-up group data, such as cohort data.

### Confidence Interval

The proposed estimator *PRR* is the ratio of two odds.

On estimating the odds ratio as $eOR={m}_{11}\cdot ({m}_{0}-{m}_{10})\cdot {m}_{10}^{-1}\cdot {({m}_{1}-{m}_{m11})}^{-1}$, the following *eSE*(*ln eOR*) is known as the maximum likelihood estimator for the standard deviation of ln *eOR* ^13^:

41 $$eSE({\rm{l}}{\rm{n}}eOR)=\sqrt{\frac{1}{{m}_{11}}+\frac{1}{{m}_{10}}+\frac{1}{{m}_{1}-{m}_{11}}+\frac{1}{{m}_{0}-{m}_{10}}}.$$

Let us apply this formula to *PRR* for estimating confidence interval (CI).

When these two odds are nonzero, the estimator of the standard deviation of the logarithm of *PRR* will be

42 $$eSE(\mathrm{ln}\,PRR)=\sqrt{\frac{1}{l-{l}_{1}}+\frac{1}{{l}_{1}}+\frac{1}{{m}_{11}}+\frac{1}{{m}_{1}-{m}_{11}}}\mathrm{.}$$

Thus, the following formulas would provide the 100(1 − *α*)% confidence limits for *PRR*.

43 $$LCL=\exp (\mathrm{ln}\,PRR-{Z}_{\alpha \mathrm{/2}}\cdot \sqrt{\frac{1}{l-{l}_{1}}+\frac{1}{{l}_{1}}+\frac{1}{{m}_{11}}+\frac{1}{{m}_{1}-{m}_{11}}})$$

and

44 $$UCL={\rm{e}}{\rm{x}}{\rm{p}}({\rm{l}}{\rm{n}}PRR+{Z}_{\alpha \mathrm{/2}}\cdot \sqrt{\frac{1}{l-{l}_{1}}+\frac{1}{{l}_{1}}+\frac{1}{{m}_{11}}+\frac{1}{{m}_{1}-{m}_{11}}}),$$

where *LCL* and *UCL* are the lower and upper limits of CI and *Z*_*α*/2_ represents the *α*/2 point of the normal distribution, such as 1.96 for 95% interval.

To prove this estimators for CI, computer simulation was conducted. It is assumed that 30% of the population 100000 was exposed. The total number of exposed and unexposed people for whom an event occurred was determined by using two sets of risks, in which the relative risk is 3: *π*_1_ = 0.03 and *π*_0_ = 0.01 or *π*_1_ = 0.3 and *π*_0_ = 0.1. Samples, exposed case-groups, and unexposed case-groups were picked from the corresponding people based on each six sets of the observed proportions, and the CI was computed each time. Each set of six proportions was chosen so that each group should be close to the size used generally in research.

Table 3 demonstrates the number of times the true relative risk was included in the 95% CI in each one million trials. It is shown that the true value (relative risk: 3) is included at a rate of approximately 95%; this method will well estimate CI.

**Table 3:** Number of times the true value (relative risk: 3.0) was included in 95% confidence interval in each one million trials.

Observed Proportion		Theoretical Number of Exposed Subjects/Total Subjects		Number of Times Including True Value	Rate
Sample	Case Group	Sample	Case Group		
A. (*π*_1_ = 0.03, *π*_0_ = 0.01)					
0.01	0.20	300/1000	180/320	953646	95.4%
0.01	0.10	300/1000	90/160	953074	95.3%
0.01	0.01	300/1000	18/32	955724	95.6%
0.10	0.20	3000/10000	180/320	969840	97.0%
0.10	0.10	3000/10000	90/160	961068	96.1%
0.10	0.01	3000/10000	18/32	958187	95.8%
B. (*π*_1_ = 0.3, *π*_0_ = 0.1)					
0.01	0.020	300/1000	180/320	938895	93.9%
0.01	0.010	300/1000	90/160	943709	94.4%
0.01	0.002	300/1000	18/32	953717	95.4%
0.10	0.020	3000/10000	180/320	951479	95.1%
0.10	0.010	3000/10000	90/160	951707	95.2%
0.10	0.002	3000/10000	18/32	956232	95.6%

For a population of 100000, in which 30000 people was exposed, two sets of risk (A and B) were applied. In A, risk of exposed subjects (*π*_1_) is 0.03 and that of unexposed subjects (*π*_0_) is 0.03; the number of exposed and unexposed subjects for whom an event occurred is 900 and 700. In B, *π*_1_ = 0.3 and *π*_0_ = 0.1; 9000 exposed subjects and 7000 unexposed subjects developed an event. Sample, exposed case group, and unexposed case group were picked one million times for each of six sets of observed proportions from the corresponding subjects, and confidence limits were computed each time.

### Application to Real-Life Data

The suicide rate among the youth of Japan is considerably high and suicide accounts for nearly half of the causes of death among those in their twenties^14^. Meanwhile, unemployment is suggested to increase suicide risk^2, 15^.

The proposed equation was applied to the latest suicide and employment data in Japan as real-life data, and confidence intervals at 95% were also estimated. The prevalence of suicide and employment among individuals in their twenties in 2015 was obtained from a statistics report published by the Ministry of Health, Labour and Welfare^16^ and the Labour Force Survey^17^. The data used are presented in Table 4. Suicide victims who were unemployed are treated as “No occupation”. Although the Labour Force Survey was conducted in a specific month in 2015 using random sampling, the indicators should represent the characteristics of the Japanese population in that year.

**Table 4:** Employment (A) and suicide rate (B) among population aged 20–29 years in Japan, 2015.

A: Employment situation			B: Incidence of suicide		
(million)	Women	Men	(real number)	Women	Men
**Total population**	**6.21**	**6.56**	**Total**	**621**	**1731**
Labour force	4.63	5.33	Self-employed or family workers	3	35
*Employed person* ^*a*^	*4.40*	*5.02*	Employees or office workers	238	892
*Unemployed person* ^*a*^	*0.23*	*0.30*	Students or pupils	82	307
Not in Labour force	1.57	1.23	No occupation	290	467
*Attending school* ^*b*^	*0.77*	*1.01*	(Unemployed)^*c*^	(19)	(62)
*Housekeeping* ^*b*^	*0.68*	*0.03*	Unknown	8	30
*Other* ^*b*^	*0.11*	*0.20*			

Under “A: Employment situation”, the population is divided into “Labour force” and “Not in labour force”. “Labour force” consists of “Employed person” and “Unemployed person” and “Not in labour force” includes “Attending school”, “Housekeeping”, and “Other”. Under “B: Incidence of suicide”, suicide victims are divided into five groups: “Self-employed or family workers”, “Employees or office workers”, “Students or pupils”, “No occupation”, and “Unknown”. In B, “Unemployed” is treated as a part of “No occupation”.

^*a*^Labour force. ^*b*^Not in Labour force. ^*c*^No occupation.

The estimation of relative risk for unemployed women is

45 $$PRR=\frac{\mathrm{(6.21}-\mathrm{0.23)}\times 1\,000\,000}{0.23\times 1\,000\,000}\times \frac{19}{621-19}=\mathrm{0.82,}$$

and the 95% confidence interval for this relative risk can be estimated as follows:

46 $$LCL=\exp (\mathrm{ln}\,0.82-1.96\cdot \sqrt{\frac{1}{\mathrm{(6.21}-\mathrm{0.23)}\times 1\,000\,000}+\frac{1}{0.23\times 1\,000\,000}+\frac{1}{19}+\frac{1}{621-19}})=0.52$$

and

47 $$UCL=\exp (\mathrm{ln}\,0.82+1.96\cdot \sqrt{\frac{1}{\mathrm{(6.21}-\mathrm{0.23)}\times 1\,000\,000}+\frac{1}{0.23\times 1\,000\,000}+\frac{1}{19}+\frac{1}{621-19}})=\mathrm{1.30.}$$

The estimation for men can be done in the same way. Thus, the estimated relative risk is 0.82 (95% CI: 0.52–1.30) for women and 0.78 (95% CI: 0.60–1.00) for men. Unemployment did not increase the risk of suicide.

Incidentally, the proportions of victims who were classified under “No occupation” are comparatively large for both women and men, and thus, the situation of no occupation might increase risk. Let us, on trial, assume that a person who is neither employed nor attending school is the same as an individual with no occupation. The number of women in no occupation is then 1.04 million (6.21 − 4.40 − 0.77 = 1.04); the estimates of the relative risk and confidence limits for women in no occupation can be computed as follows:

48 $$PRR=\frac{\mathrm{(6.21}-\mathrm{1.04)}\times 1\,000\,000}{1.04\times 1\,000\,000}\times \frac{290}{621-290}=\mathrm{4.36,}$$

49 $$LCL=\exp (\mathrm{ln}\,4.36-1.96\cdot \sqrt{\frac{1}{\mathrm{(6.21}-\mathrm{1.04)}\times 1\,000\,000}+\frac{1}{1.04\times 1\,000\,000}+\frac{1}{290}+\frac{1}{621-290}})=3.72$$

and

50 $$UCL=\exp (\mathrm{ln}\,4.36+1.96\cdot \sqrt{\frac{1}{\mathrm{(6.21}-\mathrm{1.04)}\times 1\,000\,000}+\frac{1}{1.04\times 1\,000\,000}+\frac{1}{290}+\frac{1}{621-290}})=\mathrm{5.10.}$$

For men, the number is 0.53 million (6.56 − 5.02 − 1.01 = 0.53); the estimation can be done in the same way. Thus, the relative risk would be estimated to be 4.36 (95% CI: 3.72–5.10) for women and 4.20 (95% CI: 3.78–4.67) for men.

Although the calculations were not adjusted and the definition of no occupation is tentative, these results suggest that being neither employed nor educated may substantially increase the risk of suicide among the young Japanese population. It might be also suggested that the Japanese governments should consider the indicator of unemployment.

Note that relative risks were estimated without a fresh cohort study, which is generally difficult to conduct.

## Discussion

Evaluating a change in risk of an event occurring caused by exposure to (or the presence of/occupation as) a factor is generally attempted in many research fields, such as epidemiology, medicine, social science, politics, and product development. Relative risk, which is the ratio of the risks, can be easily interpreted and widely used, but has been believed to require large-scale epidemiological research or a smaller cohort study designed for the estimation. A case control study, which compares the case and control group, is more convenient than the cohort study, but relative risk cannot be estimated using case control data. The estimator of the odds ratio, which can be calculated using case control data, is often used instead of relative risk, because the former can sometimes approximate the latter. A method to calculate relative risk using the odds ratio was also proposed. Unfortunately, the odds ratio may be misleading to interpret the change in risk and calculating relative risk using the ratio still requires either estimator of risks. Furthermore, control group data are still required, burdening researchers in terms of cost and effort.

In this study, introducing the observed proportion, an observational inconsistency preventing relative risk from being estimated in case control studies was clarified as a mathematical expression; by excluding this inconsistency, a new equation that estimates relative risk using case data was proposed. The proposed equation, which serves as an estimator of relative risk itself without approximation, requires only the exposure odds of a case group and that of all subjects to be studied; no control group is then needed. The calculation is done without using risk estimators, and thus, cohorts are also not needed. Therefore, evaluating a change in risk can be easily conducted without additional costs, efforts, and time generally needed in a fresh study. Moreover, the proposed equation was derived without using the Bayesian probabilities nor the Bayes’ theorem and is free from researcher’s resistance toward the Bayesian methods.

A method of estimating confidence limits of the proposed estimator was also presented and proved to estimate that successfully. Although there may be a more appropriate estimation method of confidence interval, pursuing the best method is beyond the scope of this paper.

Once the exposed proportions by various characteristics are investigated, changes in every risk associated with the exposure will able to be estimated by applying the proposed equation to appropriate case group data. Even the estimation of a change in risk, which has been believed to be impossible, can be done, such as the adverse effect of a social situation on the suicide rate, the effect of a policy on birthrate, or the impact of a new drug for a pandemic on survival rate. There are two caveats: the case group must comprise subjects from whom the exposed proportion was computed and the exposure to the factor must precede the occurring event. Existing statistical methods, such as adjusting confounding factors, should be also applicable for the proposed estimator.

Although the proposed equation is quite simple, its advantages will not only reduce the costs of epidemiological studies but may also make itself a powerful tool in almost all research fields that treat risks.
